# From “Can I Handle This?” to “Is It Worth It?”: The Interplay of Experience & Psychological Barriers in Tech Adoption

**DOI:** 10.1093/geroni/igaf122.3939

**Published:** 2025-12-31

**Authors:** Joonhyeog Park, Jinbo Niu, Tamara Cadet

**Affiliations:** University of Pennsylvania, Philadelphia, Pennsylvania, United States; University of Southern California, Los Angeles, California, United States; University of Pennsylvania, Philadelphia, Pennsylvania, United States

## Abstract

Assistive smart home technologies (SHTs), such as motion sensors and voice assistants, may enhance independence for older adults living at home. However, the decision to use these devices involves a complex trade-off between the benefits they offer and legitimate concerns about privacy and personal autonomy. Guided by the Unified Theory of Acceptance and Use of Technology to assess behavioral intention and its key predictors, such as usefulness and social influence, we aimed to understand how psychological barriers influence the intention to use SHTs and whether this behavior changes based on a user’s prior experience with smart devices. We analyzed online survey data from 421 U.S. adults aged 55-80 (Mage=64.5), recruited via the CloudResearch Survey Platform. The analysis employed hierarchical multivariate regression models with robust standard errors. Findings suggested for inexperienced users, the importance of both ease of use and social influence grew in decision-making as their privacy concerns increased (p<.10). Furthermore, the need for an easy-to-use system became more critical as their fear of dependency grew (p<.10). For experienced users, technology’s usefulness became the key factor in overcoming their fear of dependency (p<.05), while social influence was most critical in mitigating privacy concerns (p<.05). These findings suggest the need for tailored support in decision-making about SHTs. Inexperienced users should receive clear demonstrations of ease of use to alleviate dependency concerns, while also leveraging social proof to address their anxieties. For experienced users, support should include evidence of the technology’s benefits and endorsements from trusted sources to help navigate privacy implications.

